# A convenient method for preparing rigid-core ionic liquid crystals

**DOI:** 10.3762/bjoc.5.51

**Published:** 2009-10-07

**Authors:** Julien Fouchet, Laurent Douce, Benoît Heinrich, Richard Welter, Alain Louati

**Affiliations:** 1 Institut de Physique et Chimie des Matériaux de Strasbourg, UMR 7504, DMO, CNRS-Université de Strasbourg, BP 43, 23 rue du Loess, F-67034 Strasbourg Cedex 2, France; 2 Laboratoire DECOMET, UMR CNRS 7177-LC003, Université Louis Pasteur, 4 rue Blaise Pascal, 67000 Strasbourg, France; 3 Laboratoire d’Electrochimie Analytique, Ecole Nationale Superieure de Chimie de Mulhouse, 3 rue Alfred Werner, 68093 Mulhouse Cedex, France

**Keywords:** imidazolium, ionic liquid crystals, Ullman reaction

## Abstract

An efficient, solvent free method for the N-arylation of imidazole by 1-(dodecyloxy)-4-iodobenzene using Cu(II)-NaY as catalyst and K_2_CO_3_ as base is reported. By this synthetic approach, mesomorphic 3-[4-(dodecyloxy)phenyl]-1-methyl-1*H*-imidazol-3-ium iodide was synthesized in a two-step procedure, and its mesomorphism has been fully investigated by polarised optical microscopy, differential scanning calorimetry and X-ray diffraction. In addition its lamellar crystal structure, electrochemical behaviour and UV (absorption and emission) properties are reported.

## Introduction

Over the past decade extensive studies of ionic liquids (ILs) have revealed their many useful properties such as extremely low volatility, high thermal stability, non-flammability, high chemical and radiochemical stability, high ionic conductivity and wide electrochemical window [1–3]. In addition, the ILs have been used as reaction media increasing the yields of many syntheses and eliminating the hazards associated conventional solvents [4]. Thus are extremely versatile in that changes in both the cation and its counter anion can be used to finely tune their properties (for example: viscosity, melting point, polarity, hydrophilicity/hydrophobicity…). Important emerging applications include those in separation and extraction processes, and in various electrochemical devices, such as lithium ion batteries, fuel cells, and capacitors, as well as in synthesis and catalysis [1–5].

Liquid crystals are characterised by both mobility and self-organisation at the macroscopic level [6]. Almost all such mesomorphic materials are based on molecules combining two antagonistic units consisting of rigid (aromatic) and flexible (alkyl) or hydrophilic (polar heads) and hydrophobic (alkyl chains) parts. The subtle balance of their effects governs the formation of a multitude of supramolecular architectures depending on the temperature (thermotropic liquid crystals) and/or of the solvent (lyotropic liquid crystals) [7–8]. In the case of the thermotropic liquid crystals the arrangements give rise to nematic phases (molecules are aligned along an orientational axis), smectic phases (orientational/positional order in the layers) and columnar phases (orientational/positional order in the columns). The lyotropic compounds display not only lamellar and columnar organization but also hierarchical self-assembly in spheres (micelles), ribbons and fibres. These unique properties lead to their applications ranging from display technology through templating media for synthesis to biological activity (targeting and transporting of drugs and gene materials) [9].

Full convergence of the ionic liquid and liquid crystal fields could provide a vast range of materials (Ionic liquid crystals, ILCs) with novel and tunable characteristics such as those of ordered and oriented hybrid compound semiconductors exhibiting both electronic and ionic conductivity [10]. For this, the imidazolium unit is an excellent platform that can be designed to promote liquid crystalline phases and easily be doped by a large diversity of anions [11–21]. Variation of the N-substituents by Ullman coupling to extend the aromatic part is a facile means of creating this range [22–23].

**Scheme 1:**
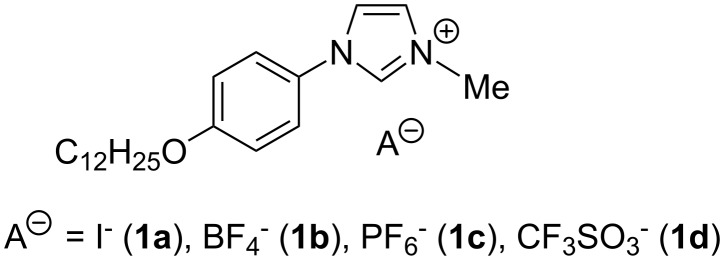
1-[4-(dodecyloxy)phenyl]-3-methyl-1*H*-imidazol-3-ium mesogenic salts.

Herein, we wish to report a solvent-free, N-arylation of imidazole as a means of expanding the aromatic core and obtaining unsymmetrical imidazolium liquid crystals (Scheme 1). We also describe the influence of the counter anion on the mesomorphism, electrochemistry and the UV properties of these imidazolium salts.

## Results and Discussion

### Synthesis and characterization

**Scheme 2:**
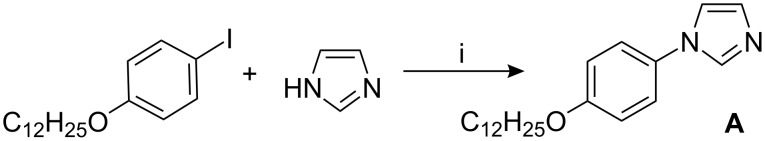
Synthesis of the imidazole **A**. Reaction conditions: (i) aryl iodide (1.37 mmol), imidazole (1.69 mmol), K_2_CO_3_ (1.51 mmol), Cu(II)NaY (148 mg), 72 h at 180 °C in a sealed tube.

Compound **1a** was obtained in a two-step procedure. The first step was a coupling reaction between 1-(dodecyloxy)-4-iodobenzene and imidazole using Cu(II)-NaY as catalyst in the presence of potassium carbonate as base [23]. The reaction took place without solvent at 180 °C in a sealed tube over 72 h to afford 1-[4-(dodecyloxy)phenyl]-1*H*-imidazole (**A**) in a good (<80%) and reproducible yield (Scheme 2). Swager has already published the synthesis of compound **A** under standard Ullman conditions (K_2_CO_3_, CuI, L-proline in DMSO, 16 h at 110 °C) [22].

**Scheme 3:**
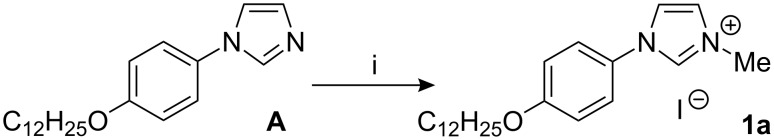
Synthesis of methyl imidazolium **1a**. Reaction conditions: (i) MeI in sealed tube, 54 h at RT.

The aryl-imidazole **A** was purified by column chromatography (ethyl acetate as eluent) on silica and characterized spectroscopically. The second step involved alkylation of **A** by iodomethane to give salt **1a** in 89% yield after purification (Scheme 3). Distinctive signals for the CH (1*H*-imidazolium) group appear in the ^1^H and ^13^C NMR spectra at 10.45 ppm and 134.97 ppm respectively.

**Figure 1:**
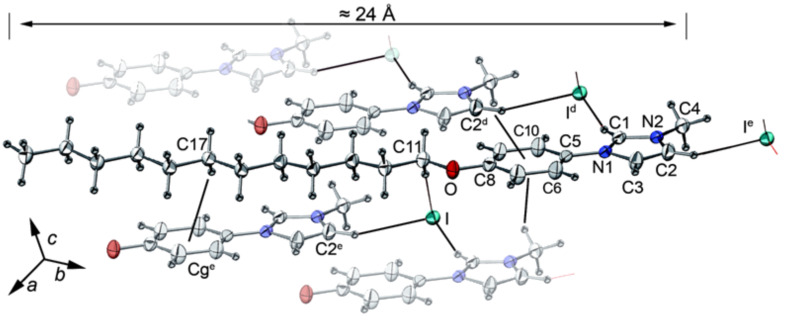
ORTEP view of compound **1a** with partial labelling. The closest molecules are represented (with lower opacity) when connected by CH-π and/or non classical H-bonds (black thin lines). The ellipsoids enclose 50% of the electronic density. Symmetry operators for equivalent positions: ^d^ = ±1+x, y, z; ^e^ = 1+x, 1+y, z.

**Figure 2:**
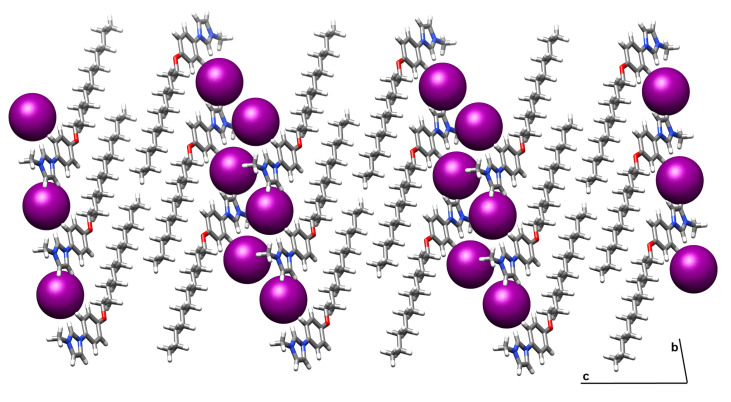
Packing diagram of compound **1a** in projection in the (b,c) lattice plane. Large spheres represent the iodine atoms.

Single crystals of **1a** suitable for X-ray diffraction were obtained by slow diffusion of ether into a CH_2_Cl_2_ solution. The compound **1a** crystallizes in the triclinic space group *P*1. A partly labelled ORTEP view showing non-classical hydrogen bonds and C-H..π interactions is given in Figure 1 (the interactions also being listed in Table 1). The alkyloxy chains are quite parallel, as is clear from the crystal packing given in Figure 2, with segregation between the rigid part (including iodine atoms) and the alkyloxy chains (≈20 Å, see Figure 2). The length of the molecule in the crystalline state is about 24 Å.

**Table 1:** Non-classical hydrogen bonds and CH..π interactions^a^ occurring in **1a**. **Cg** is the phenyl ring (C5 to C10). Symmetry operators for equivalent positions: ^d^ = ±1+x, y, z; ^e^ = 1+x, 1+y, z.

C-H..I	d_C-H_ (Å)	d_H-I_ (Å)	d_C-I_ (Å)	C-H-I (°)

C1-H1..I^d^	0.95	2.8270	3.746(5)	163.1
C2-H2..I^e^	0.95	2.9123	3.822(5)	160.6
C11-H11B..I	0.95	3.0026	3.992(5)	179.2
C-H..Cg	d_C-H_ (Å)	d_H-Cg_ (Å)	d_C-I_ (Å)	C-H-Cg (°)

C4-H4A..Cg^d^	0.95	3.109	3.502	105.6
C4-H4B..Cg^d^	0.95	3.309	3.502	93.1
C17-H17B..Cg^e^	0.95	3.310	4.207	151.5

^a^Platon software [24].

**Scheme 4:**
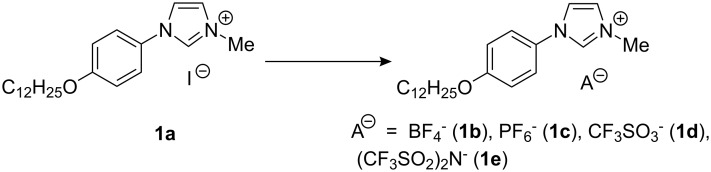
Anion metathesis in water/CH_2_Cl_2_ as solvent.

It should be emphasised that the lattice area (A = a·b·sin(γ) = 2V/d_001_ = 57.1 Å^2^) is about three times the transverse area of an all-trans crystallised chain and that even so the alkyl tails organise in segregated double layers, without interdigitation but with a tilt angle of 71° with respect to the layer normal. This large tilt angle just compensates the discrepancy between areas, maintaining the compactness of the packing and the flatness of the segregated ionic and aliphatic double layers. Apart from the crystallised state of the tails, this structure is very close to a smectic type of organisation. The segregation between the alkyl tails and the charged rigid parts indicates that by melting the chains they could show liquid crystal behaviour at a higher temperature. In order to understand the influence of the anion on the electrochemical, UV properties and mesomorphism, we prepared compounds with BF_4_^−^ (**1b**), PF_6_^−^ (**1c**), CF_3_SO_3_^−^ (**1d**) and (CF_3_SO_2_)_2_N^−^ (**1e**) anions in excellent yield by anion metathesis in water/CH_2_Cl_2_ as solvent (Scheme 4).

**Figure 3:**
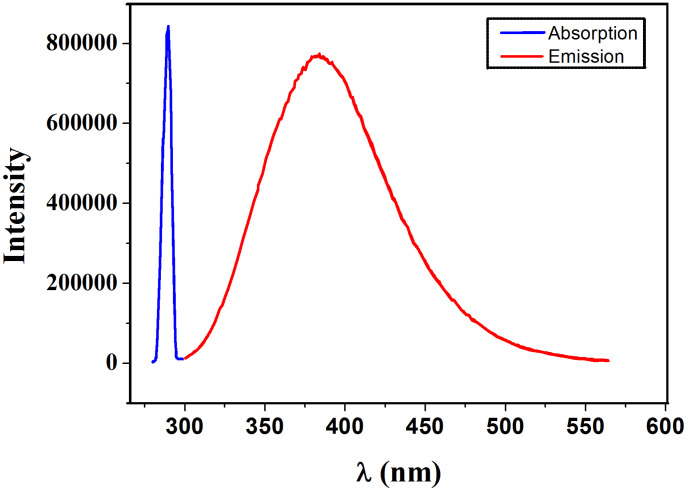
Spectra of absorption (red line) and emission (blue line) of **1a**.

All these compounds were fully characterized by ^1^H NMR, ^13^C NMR {^1^H}, FT-IR and UV spectroscopy, as well as elemental analysis. The IR spectra showed typical anion vibrations at 1024 cm^−1^ (**1b** BF_4_^−^), 826 cm^−1^ (**1c** PF_6_^−^), 1269 and 1028 cm^−1^ (**1d** CF_3_SO_3_^−^), 1358 and 1183 cm^−1^ (**1e** (CF_3_SO_2_)_2_N^−^). ^1^H NMR spectra were recorded in CDCl_3_, in which the chemical shift for the CH (1*H*-imidazolium) is very dependent upon the anion, with δ 10.45 (**1a**) 9.37 (**1b**), 9.10 (**1c**), 9.41 (**1d**) and 8.98 ppm (**1e**). This dependency is certainly due to the interactions though H-bonding and the charge localisation on the anion. The UV spectra displays typical charge transfer (π–π* or n–n*) transitions in CH_2_Cl_2_ at 240 nm (**1a** ε = 24000 M^−1^ cm^−1^), 255 nm (**1b** ε = 10500 M^−1^ cm^−1^), 249 nm (**1c** ε = 11700 M^−1^ cm^−1^), 256 nm (**1d** ε = 10100 M^−1^ cm^−1^) and 255 nm (**1e** ε = 11100 M^−1^ cm^−1^). A blue emission was also observed at 384 nm (Figure 3).

### Investigation of the Liquid Crystalline Behaviour

**Figure 4:**
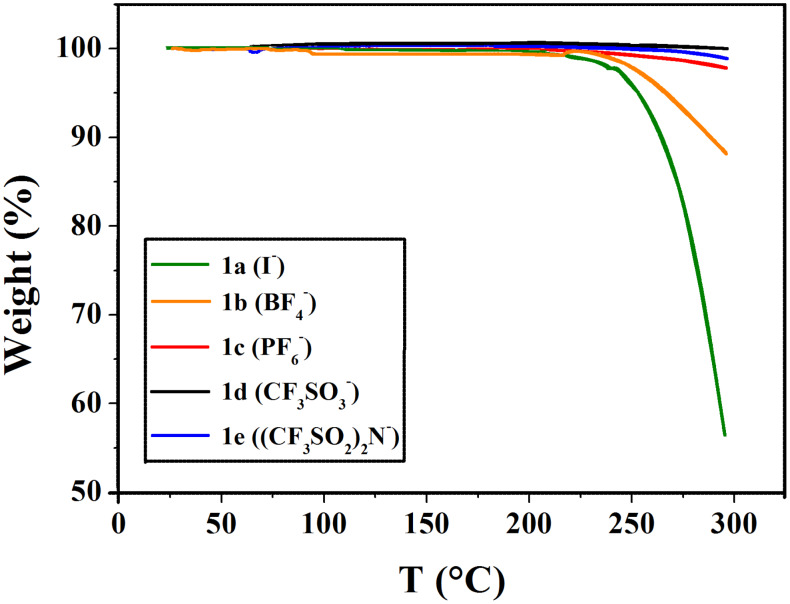
TGA measurements of the compounds **1a–e** (rate 10 °C·min^−1^, in air).

The thermogravimetric analysis of compounds **1a–e** showed the general stability order to be I^−^< BF_4_^−^ ≈ PF_6_^−^< CF_3_SO_3_^−^< (CF_3_SO_2_)_2_N^−^ (Figure 4).

**Figure 5:**
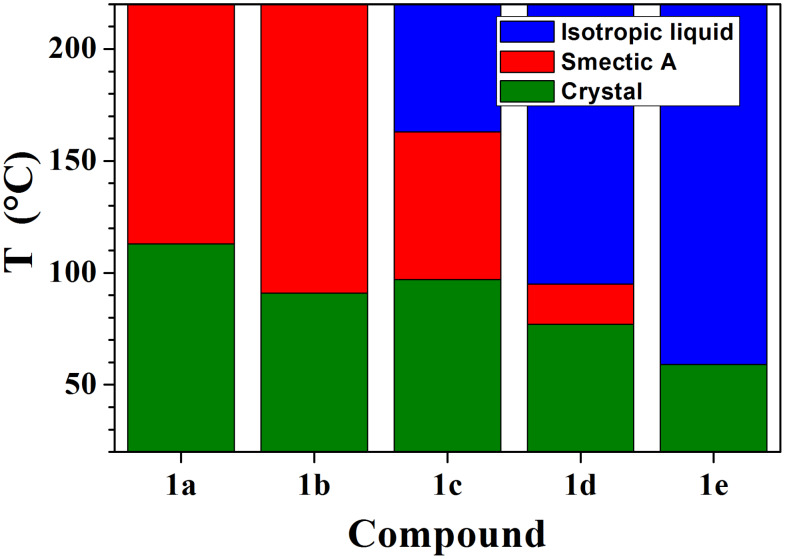
Phase transition temperatures of compounds **1a–e**.

For all the compounds, the mesomorphic behaviour and phase transition temperatures were investigated by polarized optical microscopy (POM), differential scanning calorimetry (DSC), and powder X-ray diffractometry (XRD). To avoid possible effects of hydration of the materials, all were dried in vacuo before X-ray and DSC analyses. The phase transition temperatures and the corresponding enthalpy changes derived for compounds **1a–e** are compiled in Table 2, while typical results are displayed in Figure 5.

**Table 2:** Phase transition temperatures and corresponding enthalpies determined from the 2^nd^ heating and cooling.

Anions	Phase	Temperature Cr 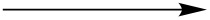 LC	Phase	Temperature LC 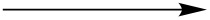 I	Phase

I^−^	Crystal	113 °C (15.83 kJ/mol) 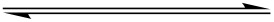 81 °C (12.76 kJ/mol)	Smectic A	250 °C 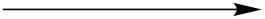	Decomposition
BF**_4_**^−^	Crystal	91 °C (30.08 kJ/mol) 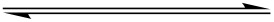 60 °C (10.62 kJ/mol)	Smectic A	230 °C 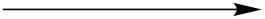	Decomposition
PF**_6_**^−^	Crystal	97 °C (36.21 kJ/mol) 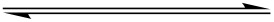 68 °C (43.34 kJ/mol)	Smectic A	163 °C (1.12 kJ/mol) 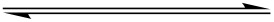 163 °C (1.76 kJ/mol)	Liquid
F**_3_**CO**_3_**^−^	Crystal	77 °C (43.28 kJ/mol) 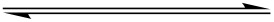 49 °C (44.00 kJ/mol)	Smectic A	95 °C (0.79 kJ/mol) 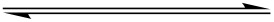 95 °C (1.04 kJ/mol)	Liquid
(F**_3_**CSO**_2_**)**_2_**N^−^	Crystal	59 °C (61.17 kJ/mol) 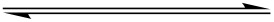 39 °C (61.12 kJ/mol)	Liquid		

Legend: Cr: Crystal, LC: Liquid Crystal, I: Isotropic Liquid.

The high stability of the compounds was also demonstrated by the absence of significant perturbation of the DSC patterns following several heating–cooling cycles. Compounds **1e**, not unexpectedly, do not show thermotropic behaviour, while the data for **1a–d** give an order of anion stabilisation of liquid crystal behaviour of Br^−^ > BF_4_^−^ > PF_6_^−^ > CF_3_SO_3_^−^ (see Figure 5).

**Figure 6:**
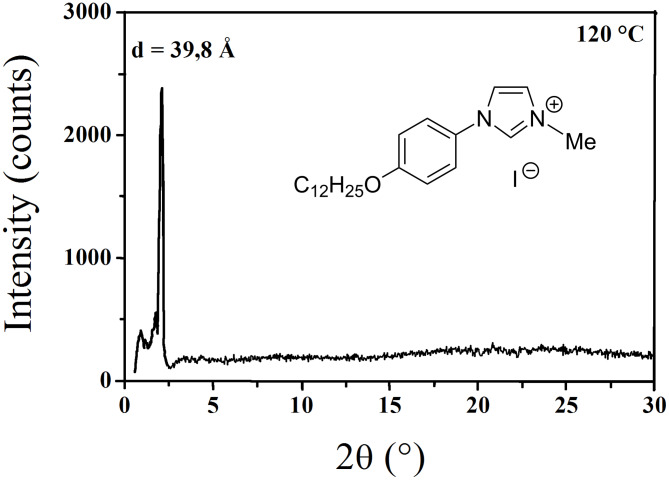
Powder X-ray diffraction pattern of compound **1a** in the liquid crystal state (T = 120 °C).

The optical textures observed during slow cooling from isotropic melt showed the emergence of a smectic A phase (appearance of Batônnet rods, turning into a wide, fan-like, focal-conic texture). The smectic structure of the liquid crystal phase was confirmed by XRD. The X-ray pattern (Figure 6) of the Smectic A form recorded at 120 °C contains a diffuse band at 4.6 Å (wide angle), which shows clearly that the alkyl chains have a liquid-like structure and are segregated from the aromatic cores.

**Figure 7:**
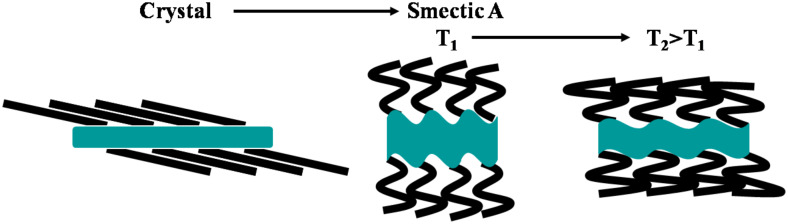
The melting process involves the ruffling of the ionic sublayer. In the smectic phase, the ruffling degree decreases with increasing temperature.

The layer thickness in the Smectic A phase was determined from the position of the sharp reflection in the small angle region (d = 39.8 Å at 120 °C) and corresponds to the alternation between the sublayer formed by the molten chains and the sublayer formed by the ionic double layer and the mesogenic parts. The thickness of the corresponding sublayers’ alternation in the crystalline phase is given by the location of the d_001_ reflection (d_001_ = 20.21 Å from single crystal pattern at room temperature). Despite the enormous difference in layer thicknesses between both phases (the extrapolation of the variation versus temperature gives d = 46.5 Å at 20 °C *i.e.* more than twice d_001_), the difference in molecular volume (smectic phase: V_mol_ = 622 Å^3^ at 20 °C; crystalline phase: V_mol_ = V/2 = 577 Å^3^) just coincides with the contribution of the chain melting [25–26], indicating that the partial volume of the ionic sublayer does not change significantly between both phases. The observed layer thickness change is therefore the consequence of the different “molecular areas” S, i.e. the projection area of a mesogen counter-ion assembly within the mean smectic plane (S = 2V_mol_/d), which is identical to the lattice area in the crystalline phase (S = V/d_001_). Thus, since no significant volume change is involved in the shrinking of S from 57.1 Å^2^ in the crystalline phase to 27 Å^2^ in the smectic A phase (value at 20 °C obtained from the extrapolation of the variation of S versus temperature), the ionic sublayer thickness d_c_ (determined as dc = 2[V_mol_/−V_ch_]/S, V_ch_ being the chain volume) simultaneously expands in proportion (from 9.5 Å in the crystalline phase to 20 Å in the smectic phase at 20 °C). These lateral shrinkage and longitudinal extension events are the result of a ruffling process of the ionic sublayers, starting from the completely flat state in the crystalline phase shown by the single crystal structure (see Figure 7).

**Figure 8:**
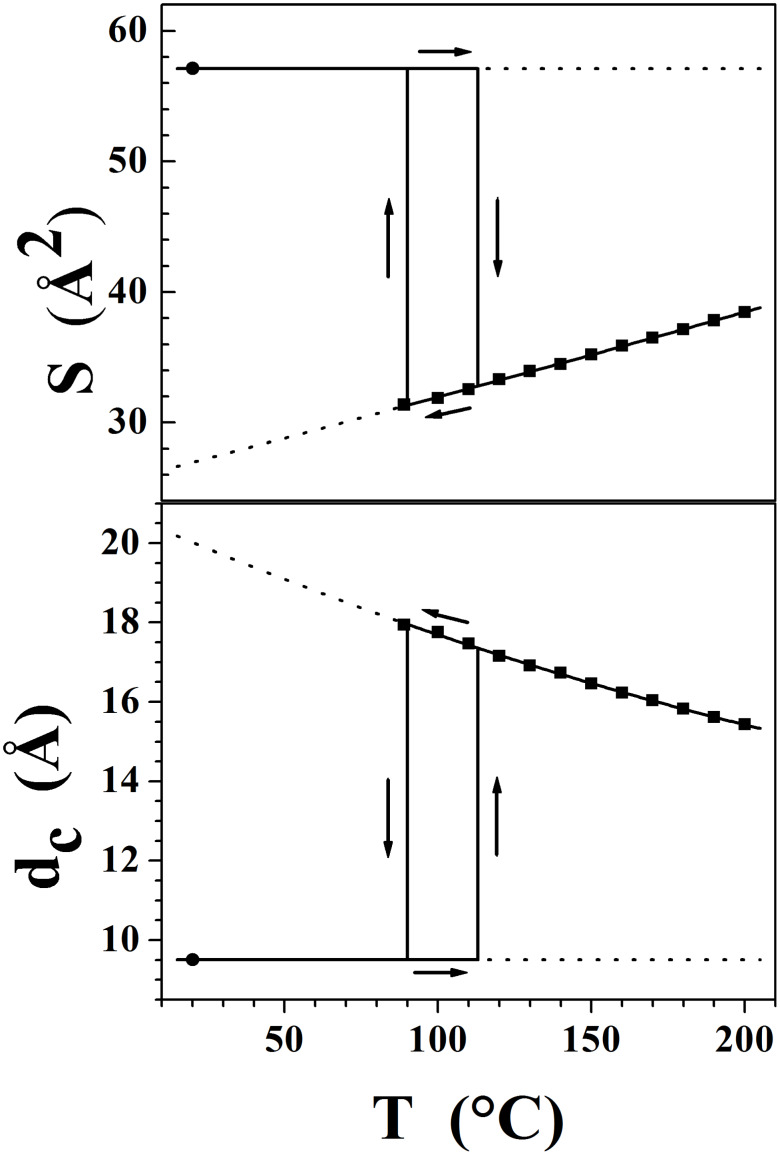
Comparison of the molecular area S and of the ionic sublayer thickness d_c_ (including mesogenic segments) in the crystalline phase (circle) and in the smectic A phase (squares) for compound **1a** (I^−^).

The maximum degree of ruffling in the smectic A phase is reached just before crystallisation, since the experimental temperature dependence of S and d_c_ indicates that the sublayers continuously spread with increasing temperature (see Figure 8).

**Figure 9:**
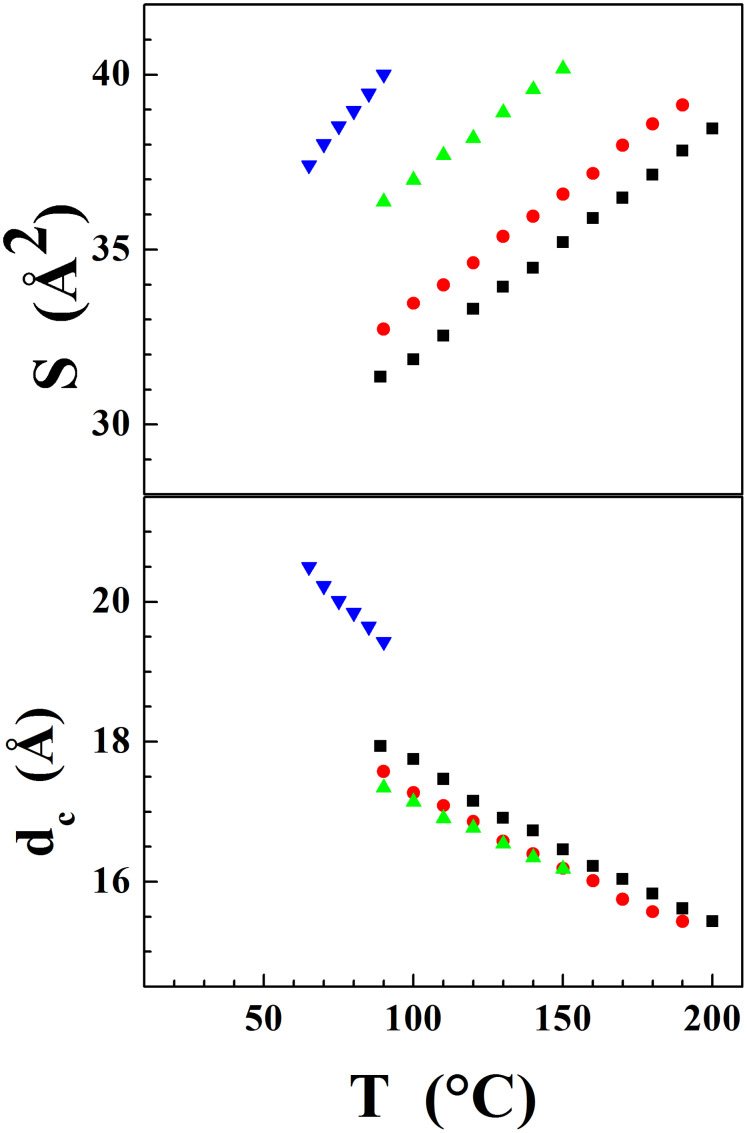
Variation with the counter-ion of the molecular area S and of the ionic sublayer thickness d_c_ (including mesogenic segments) in the smectic A phase for series 1: squares: **1a** (I^−^); circles: **1b** (BF_4_^−^); up triangles: **1c** (PF_6_^−^); down triangles: **1d** (CF_3_SO_3_^−^).

The counter-ion substitution within the series 1 involves large changes of S, but the temperature dependence and dc values are roughly the same for all terms (see Figure 9).

The influence of the substitution can therefore be considered as an anion size effect, since the lattice area expands with increasing counter-ion bulkiness without change of the degree of ruffling (the small discrepancies for compound **1d** (CF_3_SO_3_^−^) being explained by the presence of the CF_3_ lateral group, which contributes to d_c_ and perturbates slightly the interface with the aliphatic sublayer). It should be emphasised that the stability of the smectic A phase is not determined by the degree of ruffling of the ionic sublayer but by the folding degree of the tails and therefore the thickness of the aliphatic sublayers. Thus, depending upon the anion size, the isotropisation occurs at various temperatures, but for approximately the same maximum molecular area (S^max^ ≈ 41 Å^2^) and therefore similar minimum aliphatic sublayer thicknesses (d_ch_^min^ ≈ 19 Å).

To summarise, the large discrepancy between the lattice area and the cross section of the aliphatic chains are taken into account differently in the crystalline and in the smectic molecular organisations. In the crystalline phase, the ionic sublayers just impose their area and the tails tilt until dense packing is reached. In the smectic phase, tail tilting is not favourable upon the amphipathic expelling at the interface with the ionic sublayer and the system adopts a compromise molecular area associating ruffled ionic sublayers and folded aliphatic tails. With increasing temperature, the aliphatic chains spread more easily and the organisation shifts toward flat sublayers. A more detailed investigation of the molecular area variation in series involving both, counter-ion substitution and tail-length variation, has been presented elsewhere for a very similar cationic structure [20,25–26].

### Electrochemical behaviour

**Figure 10:**
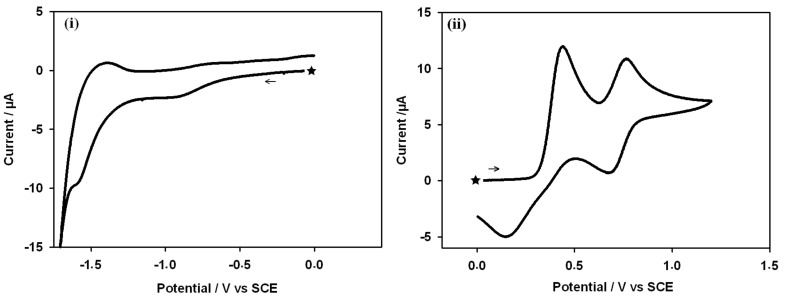
Cyclic voltammogram of **1a** in CH_3_CN (0.1 M NBu_4_PF_6_): (i), (ii) cathodic and anodic range of the voltage scan. Scan rate 100 mV·s^−1^. The black star denotes the initial and final potential.

Cyclic voltammetry was used to determine the electrochemical behaviour of the compounds **1a**, **1b** and **1c**, the voltammograms being recorded in CH_3_CN solutions containing 0.1 M NBu_4_PF_6_ as supporting electrolyte at a platinum working electrode. The peak potentials are given vs. a SCE. Representative cyclic voltammograms of **1a** are shown in Figure 10. The anodic portion of the voltage scan displays two oxidation steps having peak potentials of 0.42 V and 0.68 V, and likely involve the formation of I_2_ and possibly then a higher-oxidation-state iodine (I_3_^−^) species. As seen for **1a** (Figure 9), for **1b** and **1c** the cathodic portion of the voltage scan displays only an irreversible reduction step at ca 1.58 V, which corresponds to the reduction of the cationic imidazolium species. Note that the peak at −0.8 V is probably due to the reduction of O_2_ which is difficult to eliminate from the solution.

## Conclusion

In conclusion, we report synthetic methodology based on Ullman coupling to extend the imidazolium aromatic core. From this coupling product we have synthesized and fully characterized new mesomorphic compounds with different anions. We have also determined a structure by X-ray diffraction on a single crystal. The crystallisation shows the completely lamellar segregation between the flexible chains and the rigid part. The layers are linked to each other by the semi-interdigitation of alkyl tails. Despite an enormous difference between the cross-section of crystallised chains and the lattice area imposed by the organisation within the ionic sublayers, the latter just remain flat and the tails undergo a double layer dense packing with 71° tilting with respect to the layer normal. In the smectic phase, area matching is achieved by ruffling of the sublayers and folding of the molten aliphatic tails, the degree of ruffling decreasing with increasing temperature. The electrochemical windows have been measured and we are attempting to measure the carrier mobility in order to fully assess the prospects for using these molecules in molecular electronics. We intend also to introduce different length tails in order to obtain room temperature ionic liquid crystals, aswell as to explore use of the coupling reaction between imidazole and other aromatics and heterocycles to tune the electronic properties.

## Experimental

X-ray diffraction pattern of powder samples in Lindeman capillaries or sealed cells were measured in transmission by using a focused CuK_α1_ linear beam, temperature control being within 0.03 °C and acquisition being conducted with an Inel CS120 curved counter. The molecular volumes of all compounds were calculated with an accuracy of 0.5% from the measurements performed for an analogous compound [26] and from the methylene and counter ion partial volumes.

All reagents were purchased from commercial suppliers and used without further purification. Chromatography was carried out with Merck silica gel 60 (40–63 mm). Analytical TLC was performed with Merck silica gel 60 F254 aluminium sheets. ^1^H NMR and ^13^C NMR (300 MHz and 75 MHz respectively) spectra were recorded with a Bruker Avance 300 spectrometer at 25 °C. Chemical shifts, δ, are reported in ppm using TMS as internal standard, spin-spin coupling constants, *J*, are given in Hz and the abbreviations s, br, s, t, q, m were used to denote respectively the multiplicity of signals: singlet, broad singlet, triplet, quadruplet, multiplet. Infrared spectra were recorded (KBr pastille) with a spectrophotometer IR Digital FTS 3000. UV/Vis spectra were recorded with a spectrophotometer U-3000. Elemental analyses were performed by the analytical service at the Institut Charles Sadron and by the analytical service at the Université de Strasbourg (Strasbourg, France). The optical structures of mesophases were studied with a Leitz polarizing microscope equipped with a Mettler FP80 hot stage and an FP80 central processor. The TGA measurements were carried out on a SDTQ 600 apparatus at scanning rate of 10 °C·min^−1^. The transition temperatures and enthalpies were measured by differential scanning calorimetry with a DSC Q1000 from TA Instruments at different temperature rates (5 °C·min^−1^, 2 °C·min^−1^) on heating and cooling.

### 1-[4-(Dodecyloxy)phenyl]-1*H*-imidazole (**A**)

1-Dodecyloxy-4-iodobenzene (0.533 g, 1.37 mmol), imidazole (0.115 g, 1.69 mmol), K_2_CO_3_ (0.288 g, 1.51 mmol) and Cu(II)-NaY (0.148 g) were heated in sealed tube to 180 °C for 72 h. The reaction mixture was filtered to remove the catalyst and the filtrate was purified by column chromatography (silica gel, ethyl acetate) to afford pure **A** (0.378 g, 84%).

^1^H NMR (300 MHz, CDCl_3_): δ = 0.88 (t, 3H, *J* = 6.5 Hz, CH_3_ aliphatic chain), 1.27 (broad s, 16H, CH_2_ aliphatic chain), 1.42–1.49 (m, 2H, O-CH_2_-CH_2_-CH_2_), 1.75–1.84 (m, 2H, O-CH_2_-CH_2_), 3.98 (t, 2H, *J* = 6.6 Hz, O-CH_2_), 6.96 and 7.27 (AA′ and BB′, 2 × 2H, *J* = 9.0 Hz, CH phenyl), 7.17–7.19 (m, 2H, N-CH-CH-N), 7.75 (broad s, 1H, N-CH-N). ^13^C NMR (75 MHz, CDCl_3_): δ = 14.03 (CH_3_ aliphatic), 22.61, 25.94, 29.13, 29.27, 29.30, 29.50, 29.52, 29.57, 29.59, 31.84 (CH_2_ aliphatic), 68.40 (O-CH_2_), 115. 39 (CH phenyl), 118.67 (CH imidazolium), 123.09 (CH phenyl), 129.97 (CH imidazolium), 130.47 (N-C phenyl), 135.79 (CH imidazolium), 158.48 (C-O-CH_2_ phenyl). ν_max_/cm^−1^ 3118 (C-H aromatic), 2921 and 2851 (C-H aliphatic), 1520 (C=C aromatic), 1243 (C-O aromatic). UV/Vis (CH_2_Cl_2_): λ_max_ (ε, L·mol^−1^·cm^−1^) = 241 nm (15000). Elemental analysis for C_21_H_32_N_2_O, Cacld: C, 76.78; H, 9.82; N, 8.53%. Found: C, 76.96; H, 10.58; N, 8.57%.

### 1-[4-(Dodecyloxy)phenyl]-3-methyl-1*H*-imidazol-3-ium iodide (**1a**)

A mixture of **A** (1.069 g, 3.25 mmol) and iodomethane (2 mL, 31.80 mmol) was stirred in a sealed tube for 54 h and was heated to 40 °C for 10 minutes. Diethyl ether was added and the reaction mixture was filtered and the solid was washed with diethyl ether. Crystallization with dichloromethane and diethyl ether gave de **1a** (1.318 g, 89%).

^1^H NMR (300 MHz, CDCl_3_): δ = 0.89 (t, 3H, *J* = 6.9 Hz, CH_3_ aliphatic chain), 1.28 (broad s, 16H, CH_2_ aliphatic chain), 1.41–1.48 (m, 2H, O-CH_2_-CH_2_-CH_2_), 1.76–1.85 (m, 2H, O-CH_2_-CH_2_), 3.99 (t, 2H, *J* = 6.6 Hz, O-CH_2_), 4.27 (s, 3H, N-CH_3_), 7.04 and 7.66 (AA′ and BB′, 2 × 2H, *J* = 9.1 Hz, CH phenyl), 7.46–7.48 (m, 2H, N-CH-CH-N), 10.45 (broad s, 1H, N-CH-N). ^13^C NMR (75 MHz, CDCl_3_): δ = 14.01 (CH_3_ aliphatic), 22.58, 25.89, 29.00, 29.25, 29.29, 29.48, 29.51, 29.54, 29.57, 31.82 (CH_2_ aliphatic), 37.57 (N-CH_3_), 68.65 (O-CH_2_), 115. 99 (CH phenyl), 121.03 (CH imidazolium), 123.65 (CH phenyl), 124.42 (CH imidazolium), 127.05 (N-C phenyl), 135.49 (CH imidazolium), 160.47 (C-O-CH_2_ phenyl). ν_max_/cm^−1^ 3131 (C-H aromatic), 2921 and 2851 (C-H aliphatic), 1514 (C=C aromatic), 1251 (C-O aromatic). UV–vis (CH_2_Cl_2_): λ_max_ (ε, L·mol^−1^·cm^−1^) = 240 nm (24000). Elemental analysis for C_22_H_35_IN_2_O·1/4H_2_O, Calcd: C, 55.64; H, 7.53; N, 5.90%. Found: C, 55.78; H, 7.48; N, 5.34%.

### General procedure for metathesis in water–anion exchange

A mixture of **1a** dissolved in dichloromethane (4 mL) and a mixture of the corresponding salts dissolved in water (3 mL) were stirred together for 140 h. The organic layer was separated off, washed with water and dried over calcium chloride. Crystallization with dichloromethane and diethyl ether gave the corresponding imidazolium salt.

### 1-[4-(Dodecyloxy)phenyl]-3-methyl-1*H*-imidazol-3-ium tetrafluoroborate (**1b**)

Following the general procedure using **1a** (0.797 g, 1.69 mmol) and sodium tetrafluoroborate (0.511 g, 4.56 mmol) provided **1b** with a yield of 72% (0.525 g, 1.22 mmol).

^1^H NMR (300 MHz, CDCl_3_): δ = 0.86 (t, 3H, *J* = 6.3 Hz, C*H*_3_ aliphatic chain), 1.28 (broad s, 16H, C*H*_2_ aliphatic chain), 1.41–1.46 (m, 2H, O-CH_2_-CH_2_-C*H*_2_), 1.75–1.84 (m, 2H, O-CH_2_-C*H*_2_), 3.98 (t, 2H, *J* = 6.3 Hz, O-C*H*_2_), 4.11 (s, 3H, N-C*H*_3_), 7.02 and 7.53 (AA′ and BB′, 2 × 2H, *J* = 9.0 Hz, C*H* phenyl), 7.48 (broad s, 2H, N-C*H*-C*H*-N), 9.37 (broad s, 1H, N-C*H*-N). ^13^C NMR (75 MHz, CDCl_3_): δ = 14.07 (*C*H_3_ aliphatic), 22.65, 25.95, 29.08, 29.32, 29.37, 29.55, 29.59, 29.61, 29.64, 31.89 (*C*H_2_ aliphatic), 36.81 (N-*C*H_3_), 68.65 (O-*C*H_2_), 115. 96 (*C*H phenyl), 121.34 (*C*H imidazolium), 123.59 (*C*H phenyl), 124.50 (*C*H imidazolium), 127.24 (N-*C* phenyl), 134.97 (*C*H imidazolium), 160.47 (*C*-O-CH_2_ phenyl). ν_max_/cm^−1^ 2917 and 2849 (C-H aliphatic), 1514 (C=C aromatic), 1249 (C-O aromatic), 1024 (BF_4_^−^). UV–vis (CH_2_Cl_2_): λ_max_ (ε, L·mol^−1^·cm^−1^) = 255 nm (10500). Elemental analysis for C_22_H_35_BF_4_N_2_O·3/4H_2_O, Calcd: C 59.53, H 8.29, N 6.31%. Found: C 59.74, H 8.02, N 6.20%.

### 1-[4-(Dodecyloxy)phenyl]-3-methyl-1*H*-imidazol-3-ium hexafluorophosphate (**1c**)

Following the general procedure using **1a** (0.695 g, 1.48 mmol) and potassium hexafluoroborate (0.518 g, 2.18 mmol) provided **1c** with a yield of 84% (0.607 g, 1.24 mmol).

^1^H NMR (300 MHz, CDCl_3_): δ = 0.89 (t, 3H, *J* = 6.8 Hz, C*H*_ 3_ aliphatic chain), 1.28 (broad s, 16H, C*H*_2_ aliphatic chain), 1.41–1.46 (m, 2H, O-CH_2_-CH_2_-C*H*_2_), 1.76–1.85 (m, 2H, O-CH_2_-C*H*_2_), 3.98 (t, 2H, *J* = 6.6 Hz, O-C*H*_2_), 4.07 (s, 3H, N-C*H*_3_), 7.02 and 7.48 (AA′ and BB′, 2 × 2H, J = 8.8 Hz, C*H* phenyl), 7.45 (broad s, 2H, N-C*H*-C*H*-N), 9.10 (broad s, 1H, N-C*H*-N). ^13^C NMR (75 MHz, CDCl_3_): δ = 14.06 (*C*H_3_ aliphatic), 22.65, 25.95, 29.08, 29.32, 29.37, 29.55, 29.59, 29.61, 29.64, 31.89 (*C*H_2_ aliphatic), 36.81 (N-*C*H_3_), 68.65 (O-*C*H_2_), 115. 91 (*C*H phenyl), 121.60 (*C*H imidazolium), 123.70 (*C*H phenyl), 124.33 (*C*H imidazolium), 127.21 (N-*C* phenyl), 134.42 (*C*H imidazolium), 160.51 (*C*-O-CH_2_ phenyl). ν_max_/cm^−1^ 2921 and 2850 (C-H aliphatic), 1516 (C=C aromatic), 1255 (C-O aromatic), 826 cm^−1^ (PF_6_^−^). UV–vis (CH_2_Cl_2_): λ_max_ (ε, L·mol^−1^·cm^−1^) = 249 nm (11700). Elemental analysis for C_22_H_35_F_6_N_2_OP·1/7H_2_O, Calcd: C 53.81, H 7.24, N 5.70%. Found: C 53.77, H 7.31, N 5.51%.

### 1-[4-(Dodecyloxy)phenyl]-3-methyl-1*H*-imidazol-3-ium trifluoromethanesulfonate (**1d**)

Following the general procedure using **1a** (0.730 g, 1.55 mmol) and sodium trifluoromethanesulfonate (0.616 g, 3.51 mmol) provided **1d** with a yield of 46% (0.349 g, 0.71 mmol).

^1^H NMR (300 MHz, CDCl_3_): δ = 0.89 (t, 3H, *J* = 6.8Hz, C*H*_3_ aliphatic chain), 1.28 (broad s, 16H, C*H*_2_ aliphatic chain), 1.41–1.46 (m, 2H, O-CH_2_-CH_2_-C*H*_2_), 1.76–1.83 (m, 2H, O-CH_2_-C*H*_2_), 3.99 (t, 2H, *J* = 6.6 Hz, O-C*H*_2_), 4.10 (s, 3H, N-C*H*_3_), 7.02 and 7.51 (AA′ and BB′, 2 × 2H, *J* = 8.8 Hz, C*H* phenyl), 7.49 (broad s, 2H, N-C*H*-C*H*-N), 9.41 (broad s, 1H, N-C*H*-N). ^13^C NMR (75 MHz, CDCl_3_): δ = 13.96 (*C*H_3_ aliphatic), 22.54, 25.85, 28.97, 29.21, 29.26, 29.44, 29.48, 29.51, 29.53, 31.78 (*C*H_2_ aliphatique), 36.52 (N-*C*H_3_), 68.54 (O-*C*H_2_), 115. 84 (*C*H phenyl), 120.49 (q, *J* = 318.18 Hz, *C*F_3_SO_3_^−^), 121.36 (*C*H imidazolium), 123.33 (*C*H phenyl), 124.44 (*C*H imidazolium), 127.15 (N-*C* phenyl), 134.95 (*C*H imidazolium), 160.36 (*C*-O-CH_2_ phenyl). ν_max_/cm^−1^ 3119 (C-H aromatic), 2915 and 2849 (C-H aliphatic), 1520 (C=C aromatic), 1269 and 1028 (CF_3_SO_3_^−^). UV–vis (CH_2_Cl_2_): λ_max_ (ε, L·mol^−1^·cm^−1^) = 256 nm (10100). Elemental analysis for C_22_H_35_F_3_N_2_O_4_S, Cacld: C 56.08, H 7.16, N 6.59%. Found: C 55.84, H 6.86, N 5.40%.

### 1-[4-(Dodecyloxy)phenyl]-3-methyl-1*H*-imidazol-3-ium bis(trifluoromethane) sulfonamide (**1e**)

**1a** (0.101 g, 0.21 mmol) and lithium bis(trifluoromethane)sulfonamide (0.145 g, 0.51 mmol) were dissolved in water (3 mL) and stirred for 140 h. The precipitate was filtred and washed. Crystallization (chloroform/cyclohexane) provided **1e** with a yield of 90% (0.121 g, 0.19 mmol).

^1^H NMR (300 MHz, CDCl_3_): δ = 0.89 (t, 3H, *J* = 6.8 Hz, C*H*_3_ aliphatic chain), 1.28 (broad s, 16H, C*H*_2_ aliphatic chain), 1.42–1.52 (m, 2H, O-CH_2_-CH_2_-C*H*_2_), 1.77–1.86 (m, 2H, O-CH_2_-C*H*_2_), 4.01 (t, 2H, *J* = 6.6 Hz, O-C*H*_2_), 4.07 (s, 3H, N-C*H*_3_), 7.05 and 7.46 (AA′ and BB′, 2 × 2H, *J* = 8.8 Hz, C*H* phenyl), 7.43–7.49 (m, 2H, N-C*H*-C*H*-N), 8.98 (broad s, 1H, N-C*H*-N). ^13^C NMR (75 MHz, CDCl_3_): δ = 14.06 (*C*H_3_ aliphatic), 22.65, 25.94, 29.05, 29.31, 29.34, 29.53, 29.57, 29.60, 29.63, 31.89 (*C*H_2_ aliphatic), 36.69 (N-*C*H_3_), 68.70 (O-*C*H_2_), 116.04 (*C*H phenyl), 119.77 (q, *J* = 319.29 Hz, *C*F_3_SO_3_^−^), 121.89 (*C*H imidazolium), 123.77 (*C*H phenyl), 124.31 (*C*H imidazolium), 126.99 (N-*C* phenyl), 134.67 (*C*H imidazolium), 160.78 (*C*-O-CH_2_ phenyl). ν_max_/cm^−1^ 2918 and 2850 (C-H aliphatic), 1517 (C=C aromatic), 1358 cm^−1^ and 1183 ((CF_3_SO_2_)_2_N^−^). UV–vis (CH_2_Cl_2_): λ_max_ (ε, L·mol^−1^·cm^−1^) = 255 nm (11100). Elemental analysis for C_24_H_35_F_6_N_3_O_5_S_2_._1/2_H_2_O, Cacld: C 45.56, H 5.74, N 6.64%. Found: C 45.52, H 5.66, N 6.58%.

## References

[1] Wasserscheid P, Welton T (2003). Ionic Liquid in Synthesis.

[2] Rogers R B, Seddon K R (2005). Ionic Liquids IIIA: Fundamentals, Progress, Challenges and Opportunities.

[3] Rogers R B, Seddon K R (2005). Ionic Liquids IIIB: Fundamentals, Progress, Challenges and Opportunities.

[4] Dupont J, de Souza R F, Suarez P A (2002). Chem Rev.

[5] Endres F, Abbott A P, MacFarlane D R (2008). Electrodeposition from Ionic liquids.

[6] Demus D, Goodby J W, Gray G W (1998). Handbook of Liquid Crystals, Vols. 1, 2a, 2b and 3.

[7] Guillon D (1999). Struct Bond.

[8] Goodby J W, Saez I M, Cowling S J, Görtz V, Draper M, Hall A W, Sia S, Cosquer G, Lee S-E, Raynes E P (2008). Angew Chem, Int Ed.

[9] Dobbs W, Heinrich B, Bourgogne C, Donnio B, Terazzi E, Bonnet M-E, Stock F, Erbacher P, Bolcato-Bellemin A-L, Douce L (2009). J Am Chem Soc.

[10] Ohno H (2005). Electrochemical Aspect of Ionic Liquids.

[11] Binnemans K (2005). Chem Rev.

[12] Bowlas C J, Bruce D W, Seddon K R (1996). Chem Commun.

[13] Yoshio M, Mukai T, Ohno H, Kato T (2004). J Am Chem Soc.

[14] Taubert A (2004). Angew Chem, Int Ed.

[15] Suisse J-M, Bellemin-Laponnaz S, Douce L, Maisse-François A, Welter R (2005). Tetrahedron Lett.

[16] Dobbs W, Suisse J-M, Douce L, Welter R (2006). Angew Chem, Int Ed.

[17] Dobbs W, Douce L, Allouche L, Louati A, Malbosc F, Welter R (2006). New J Chem.

[18] Suisse J-M, Douce L, Bellemin-Laponnaz S, Maisse-François A, Welter R, Miyake Y, Shimizu Y (2007). Eur J Inorg Chem.

[19] Yazaki S, Kamikawa Y, Yoshio M, Hamasaki A, Mukai T, Ohno H, Kato T (2008). Chem Lett.

[20] Yoshio M, Ichikawa T, Shimura H, Kagata T, Hamasaki A, Mukai T, Ohno H, Kato T (2007). Bull Chem Soc Jpn.

[21] Fanta P E (1974). Synthesis.

[22] Kouwer P H J, Swager T M (2007). J Am Chem Soc.

[23] Kantam M L, Rao B P C, Choudary B M, Reddy R S (2006). Synlett.

[24] Spek A L (2003). J Appl Crystallogr.

[25] Cruz C, Heinrich B, Ribeiro A C, Bruce D W, Guillon D (2000). Liq Cryst.

[26] Dobbs W, Heinrich B, Douce L (2009). Beilstein J Org Chem.

